# Supplementary material for: The relationship between biodiversity and wetland cover varies across regions of the conterminous United States
Source: PLoS One. 2020 May 1;15(5):e0232052. doi: 10.1371/journal.pone.0232052 (PMC7194442; doi:10.1371/journal.pone.0232052)
Supplement: S1 Appendix — (DOCX) [file pone.0232052.s001.docx]

**Posterior Sampling Algorithm & Traceplots**

**Full Conditional Distributions**

This section provides the full conditional distribution of all model parameters. We have the following likelihood function:

$$L\left( \boldsymbol{\alpha},\boldsymbol{\phi}, \boldsymbol{\psi}; \boldsymbol{Y, X, Z} \right)=\prod_{s=1}^{S} \frac{\lambda_{s}^{y_{s}}e^{-\lambda_{s}}}{y_{s}!}$$

where $S=38976$ is the number of polygon features, $\boldsymbol{Y}=(y_{1},y_{2},\ldots, y_{S})'$, $\boldsymbol{X=}diag\left( x_{1},x_{2},\ldots,x_{S} \right)$, and $\boldsymbol{Z=}\left\{ \left( 1, z_{1,1},\ldots,z_{1,P} \right)^{'}, \left( 1, z_{2,1},\ldots,z_{2,P} \right)^{'},\ldots,\left( 1, z_{S,1},\ldots,z_{S,P} \right)^{'} \right\}^{'}.$

The full conditional distributions for the model parameters are as follows:

$$f\left( \boldsymbol{\alpha};\boldsymbol{\phi}, \boldsymbol{\psi,} \boldsymbol{Y, X, Z} \right)\boldsymbol{\propto} L\left( \boldsymbol{\alpha},\boldsymbol{\phi}, \boldsymbol{\psi}; \boldsymbol{Y, X, Z} \right)\pi(\boldsymbol{\alpha};\sigma_{\alpha}^{-2})$$

$$f\left( \boldsymbol{\phi};\boldsymbol{\alpha}, \boldsymbol{\psi}, \boldsymbol{Y, X, Z} \right)\boldsymbol{\propto} L\left( \boldsymbol{\alpha},\boldsymbol{\phi}, \boldsymbol{\psi}; \boldsymbol{Y, X, Z} \right)\pi(\boldsymbol{\phi};\sigma^{-2})$$

$$f\left( \boldsymbol{\psi};\boldsymbol{\alpha, \phi}, \boldsymbol{Y, X, Z} \right)\boldsymbol{\propto} L\left( \boldsymbol{\alpha},\boldsymbol{\phi}, \boldsymbol{\psi}; \boldsymbol{Y, X, Z} \right)\pi(\boldsymbol{\psi};\tau^{-2})$$

$$f\left( \sigma^{2};\boldsymbol{\phi} \right)\boldsymbol{=}IG(Q/2 + \alpha_{\sigma},\boldsymbol{\phi}^{\boldsymbol{'}}\left( \boldsymbol{D}_{\boldsymbol{\phi}}-\boldsymbol{W}_{\boldsymbol{\phi}} \right)\boldsymbol{\phi}/2 \boldsymbol{+}\beta_{\sigma} )$$

$f\left( \tau^{2};\boldsymbol{\psi} \right)\boldsymbol{=}IG(18/2 + \alpha_{\tau},\boldsymbol{\psi}^{\boldsymbol{'}}\left( \boldsymbol{D}_{\boldsymbol{\psi}}-\boldsymbol{W}_{\boldsymbol{\psi}} \right)\boldsymbol{\psi}/2 \boldsymbol{+}\beta_{\tau})$,

where $\pi(\boldsymbol{\alpha};\sigma_{\alpha}^{-2})$, $\pi\left( \boldsymbol{\phi};\sigma^{-2} \right)$, and $\pi(\boldsymbol{\psi};\tau^{-2})$ denote the prior distributions of $\boldsymbol{\alpha}$, $\boldsymbol{\phi}$, and $\boldsymbol{\psi}$ respectively.

**MCMC Routine**

A step-by-step implementation of the MCMC posterior sampling routine is provided below:

1. Initialize $\boldsymbol{\alpha}$, $\boldsymbol{\phi}$, $\boldsymbol{\psi}$,$\sigma^{2}$ and$\tau^{2}$.
2. Set $C_{\alpha, 0}=C_{\alpha,1}=\ldots=C_{\alpha,P}=C_{\phi1}=C_{\phi,2}=\ldots=C_{\phi,Q}= C_{\psi,1}=C_{\psi,2}=\ldots=C_{\psi,18}=0$.
3. Set $\delta_{\alpha, 0}=\delta_{\alpha,1}=\ldots=\delta_{\alpha,P}=0.5$.
4. Set $\delta_{\phi,1}=\delta_{\phi,2}=\ldots=\delta_{\phi,Q}= \delta_{\psi,1}=\delta_{\psi,2}=\ldots=\delta_{\psi,18}=0$.1.
5. For $g=1, 2, \ldots, G$, do:
6. For $p=0,1, \ldots, P$, sample $\alpha_{p}$ using the following Metropolis step:
7. Sample $\alpha_{p}^{*}$ from $N(\alpha_{p},\delta_{\alpha,p}^{-2})$.
8. Set $\boldsymbol{\alpha}^{\boldsymbol{*}}\boldsymbol{=\alpha}$ with the *pth* entry equal to $\alpha_{p}^{*}$.
9. Compute $A=min\{ 1, \frac{f\left( \boldsymbol{\alpha}^{\boldsymbol{*}};\boldsymbol{\phi}, \boldsymbol{\psi}, \boldsymbol{Y, X, Z} \right)}{f\left( \boldsymbol{\alpha};\boldsymbol{\phi}, \boldsymbol{\psi}, \boldsymbol{Y, X, Z} \right)}$}.
10. Update $\boldsymbol{\alpha=}\boldsymbol{\alpha}^{\boldsymbol{*}}$ with probability *A* and set $C_{\alpha, p}= C_{\alpha, p}+1$ if $\boldsymbol{\alpha}$ was updated.
11. For $s=1, 2, \ldots, Q$, sample $\phi_{q}$ using the following Metropolis step:
12. Sample $\phi_{q}^{*}$ from $N(\phi_{q},\delta_{\phi,q}^{-2})$.
13. Set $\boldsymbol{\phi}^{\boldsymbol{*}}\boldsymbol{=\phi}$ with the *qth* entry equal to $\phi_{q}^{*}$.
14. Compute $A=min\{ 1, \frac{f\left( \boldsymbol{\alpha}; \boldsymbol{\phi}^{\boldsymbol{*}}, \boldsymbol{\psi}, \boldsymbol{Y, X, Z} \right)}{f\left( \boldsymbol{\alpha}; \boldsymbol{\phi}^{\boldsymbol{*}}, \boldsymbol{\psi}, \boldsymbol{Y, X, Z} \right)}$}.
15. Update $\boldsymbol{\phi}=\boldsymbol{\phi}^{\boldsymbol{*}}$ with probability *A* and set $C_{\phi, q}= C_{\phi, q}+1$ if $\boldsymbol{\phi}$ was updated.
16. For $r=1, 2, \ldots, 18$, sample $\psi_{r}$ using the following Metropolis step:
17. Sample $\psi_{r}^{*}$ from $N(\psi_{r},\delta_{\psi,r}^{-2})$.
18. Set $\boldsymbol{\psi}^{\boldsymbol{*}}\boldsymbol{=\psi}$ with the *rth* entry equal to $\psi_{r}^{*}$.
19. Compute $A=min\{ 1, \frac{f\left( \boldsymbol{\alpha};\boldsymbol{\phi}, \boldsymbol{\psi}^{\boldsymbol{*}}; \boldsymbol{Y, X, Z} \right)}{f\left( \boldsymbol{\alpha};\boldsymbol{\phi}, \boldsymbol{\psi}^{\boldsymbol{*}}; \boldsymbol{Y, X, Z} \right)}$}.
20. Update $\boldsymbol{\psi}=\boldsymbol{\psi}^{\boldsymbol{*}}$ with probability *A* and set $C_{\psi, r}= C_{\psi, r}+1$ if $\boldsymbol{\psi}$ was updated.
21. Sample $\sigma^{2}$from $f\left( \sigma^{2};\boldsymbol{\phi} \right)$ in a Gibbs step.
22. Sample $\tau^{2}$from $f\left( \tau^{2};\boldsymbol{\psi} \right)$ in a Gibbs step.
23. Save $\boldsymbol{\alpha}^{\boldsymbol{g}}\boldsymbol{=\alpha}$, $\boldsymbol{\phi}^{\boldsymbol{g}}\boldsymbol{=\phi}$, $\boldsymbol{\psi}^{\boldsymbol{g}}\boldsymbol{=\psi}$,$\sigma^{2g}=\sigma^{2}$, and $\tau^{2g}=\tau^{2}$.
24. If $g mod 100=0$, do:
25. For $p=0, 1, \ldots, P$, do:
26. If $C_{\alpha,p}<10$, do:
27. Set $\delta_{\alpha,p}=0.9\delta_{\alpha,p}$.
28. If $C_{\alpha,p}>75$, do:
29. Set $\delta_{\alpha,p}=1.1\delta_{\alpha,p}$.
30. For $q=1, 2, \ldots, Q$, do:

1. If $C_{\phi,q}<10$, do:

1. Set $\delta_{\phi,q}=0.9\delta_{\phi,q}$.

2. If $C_{\phi,q}>75$, do:

1. Set $\delta_{\phi,q}=1.1\delta_{\phi,q}$.
2. For $r=1, 2, \ldots, 18$, do:

1. If $C_{\psi,r}<10$, do:

1. Set $\delta_{\psi,r}=0.9\delta_{\psi,r}$.

2. If $C_{\psi,r}>75$, do:

1. Set $\delta_{\psi,r}=1.1\delta_{\psi,r}$.

4. Set $C_{\alpha, 0}=C_{\alpha,1}=\ldots=C_{\alpha,P}=C_{\phi,1}=C_{\phi,2}=\ldots=C_{\phi,Q}= C_{\psi,1}=C_{\psi,2}= \ldots=C_{\psi,18}=0$.

We note that the numerical performance of the MCMC sampling routine outlined above may be enhanced by computing the log of the acceptance probabilities (*A* terms) and then exponentiating rather that attempting to evaluate the full conditional distributions directly. It is also advisable to remove any factors common to the numerator and denominator (such as the $y_{s}!$ terms) from the full conditional distributions before evaluating the acceptable probabilities.

**Model Traceplots**

*Plots from the amphibians model (red line indicates the end of the burn-in phase):*

*
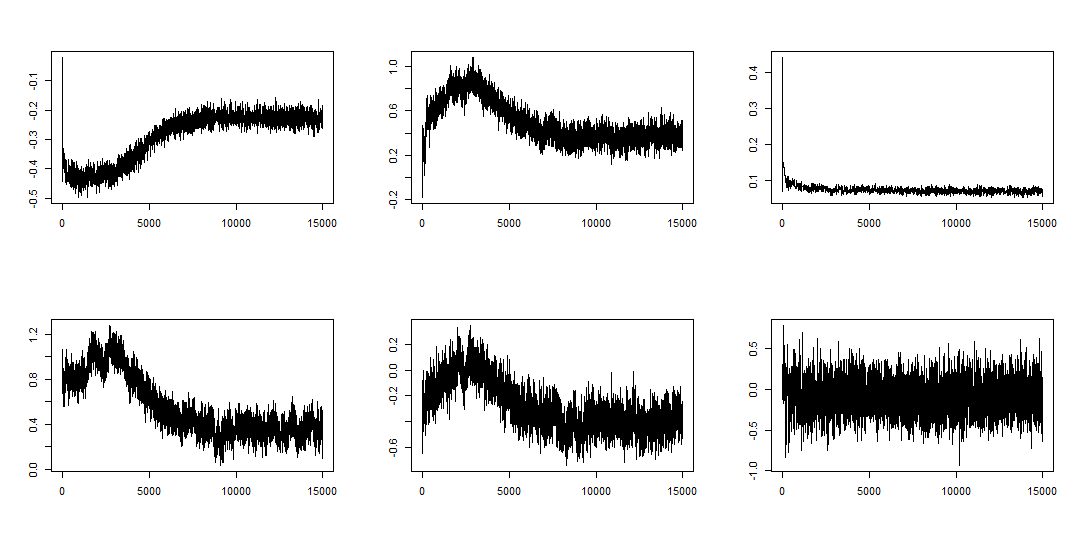
*

*
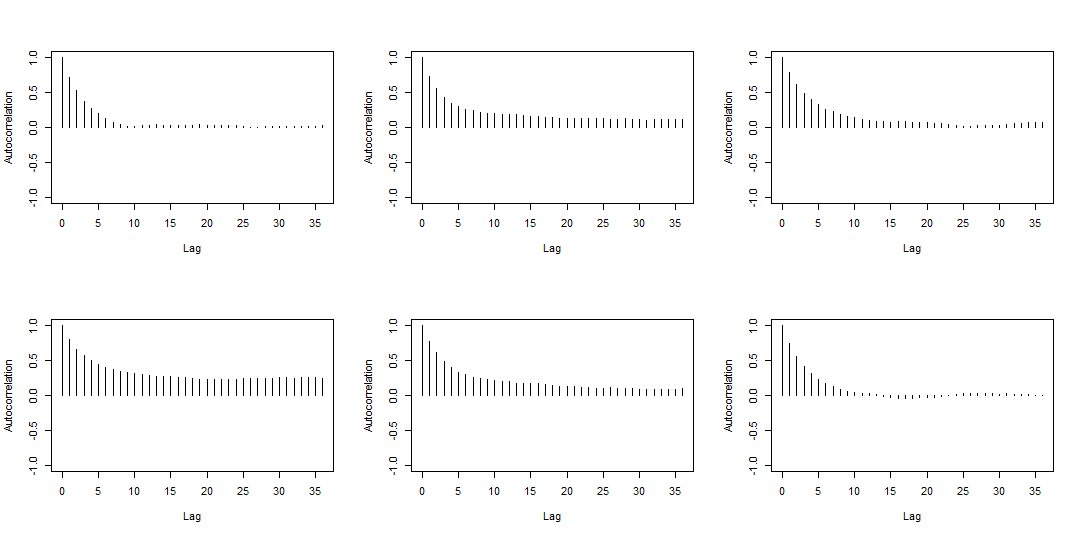
*

*Plots from the birds model:
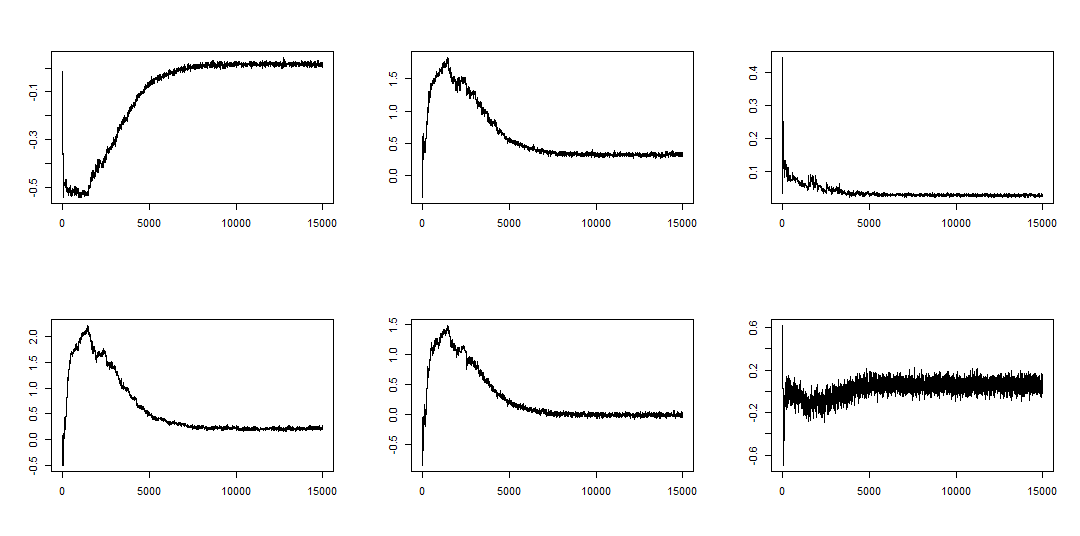

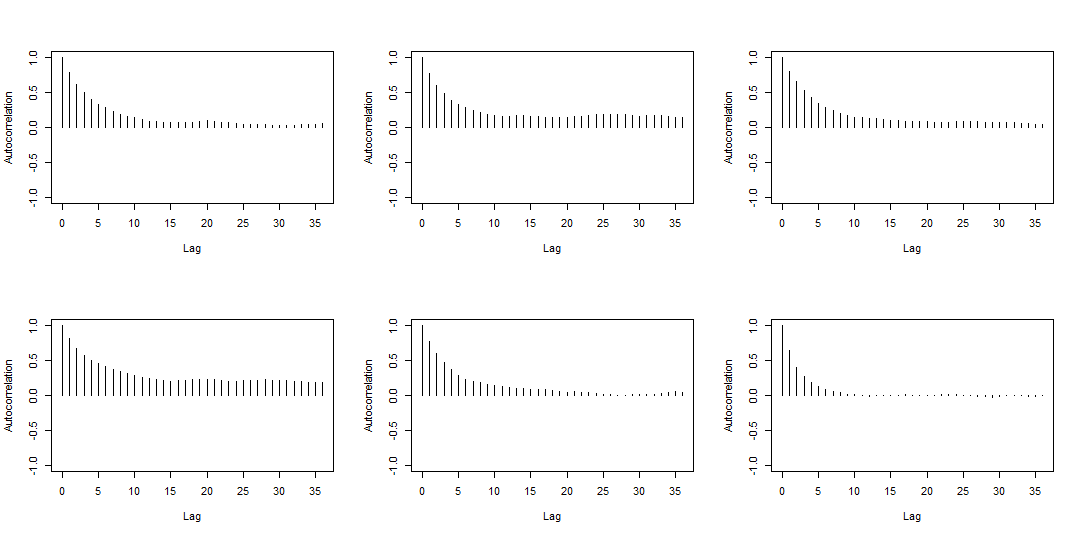
*

*Plots from the endemics model:*

*
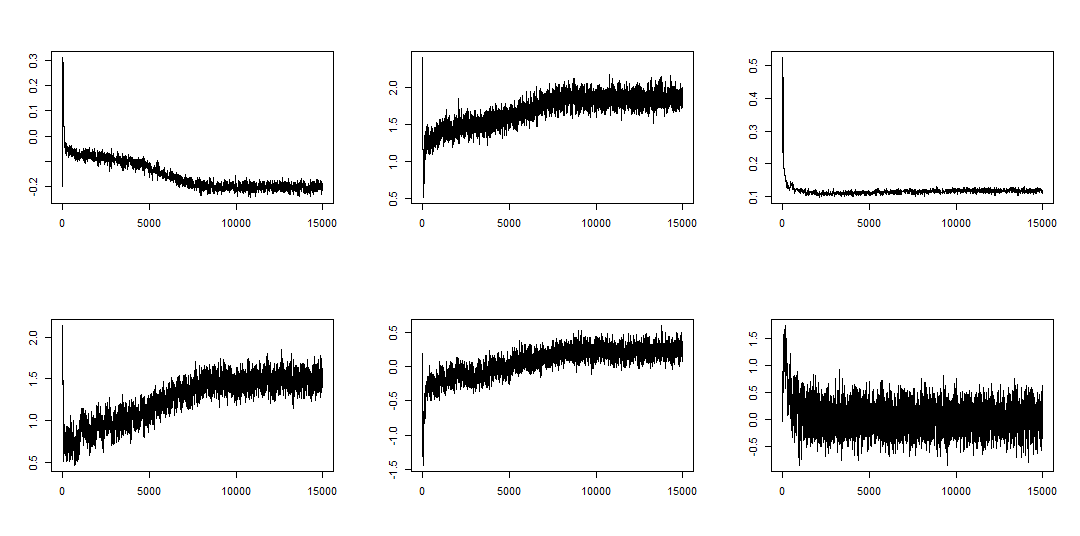

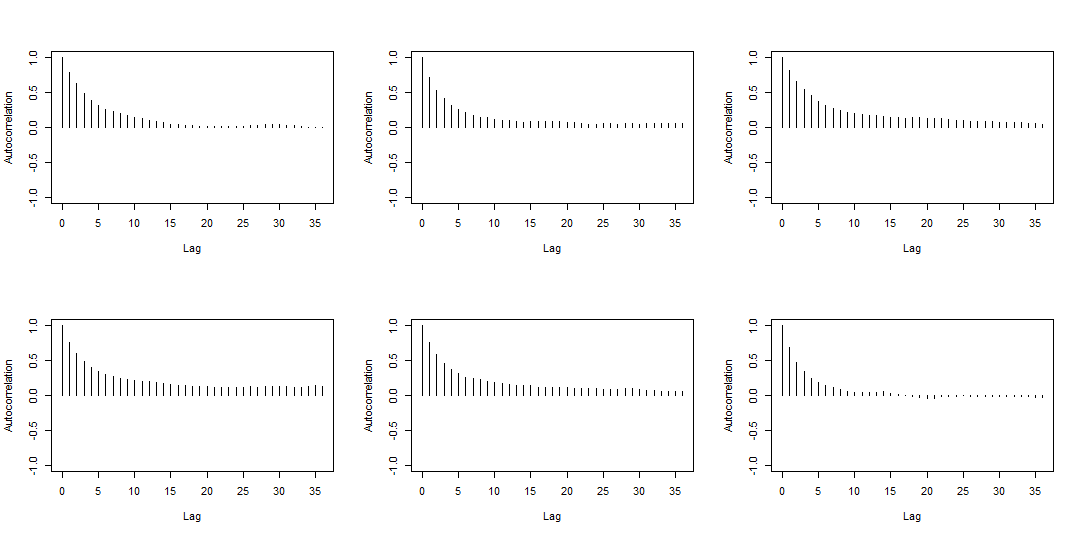
*

*Plots from the mammals model:*

*
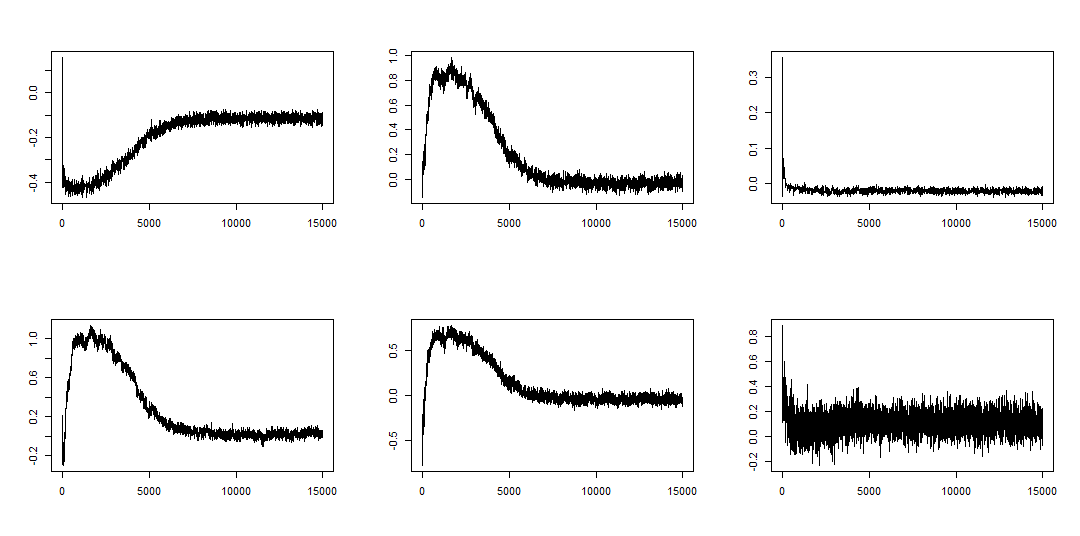

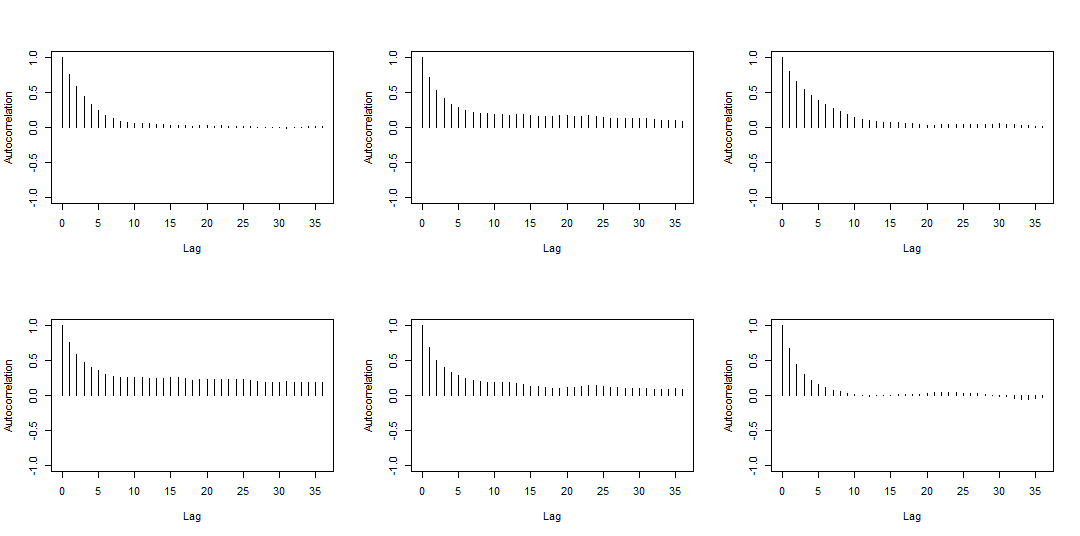
*

*Plots from the reptiles model:*

*
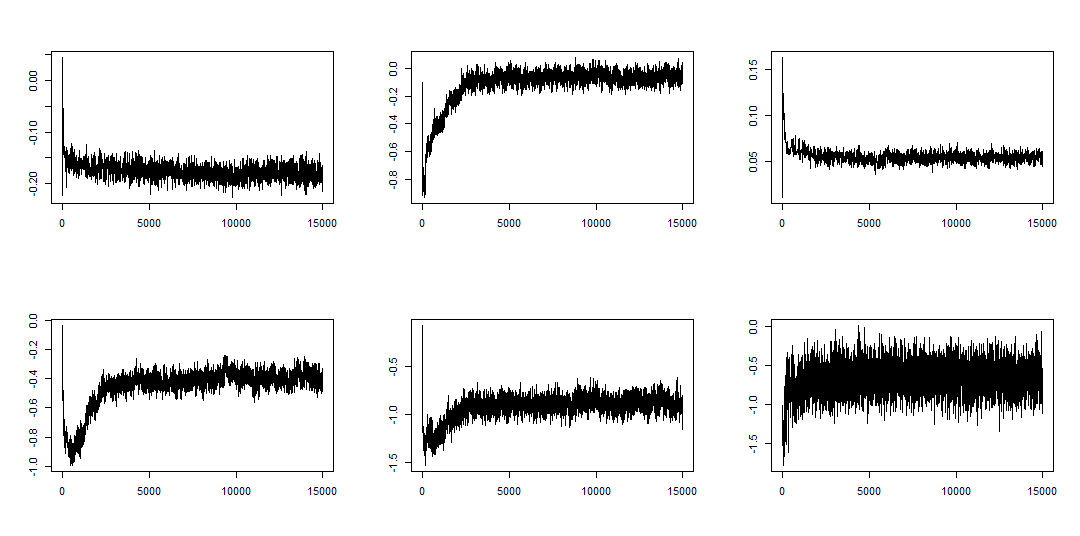

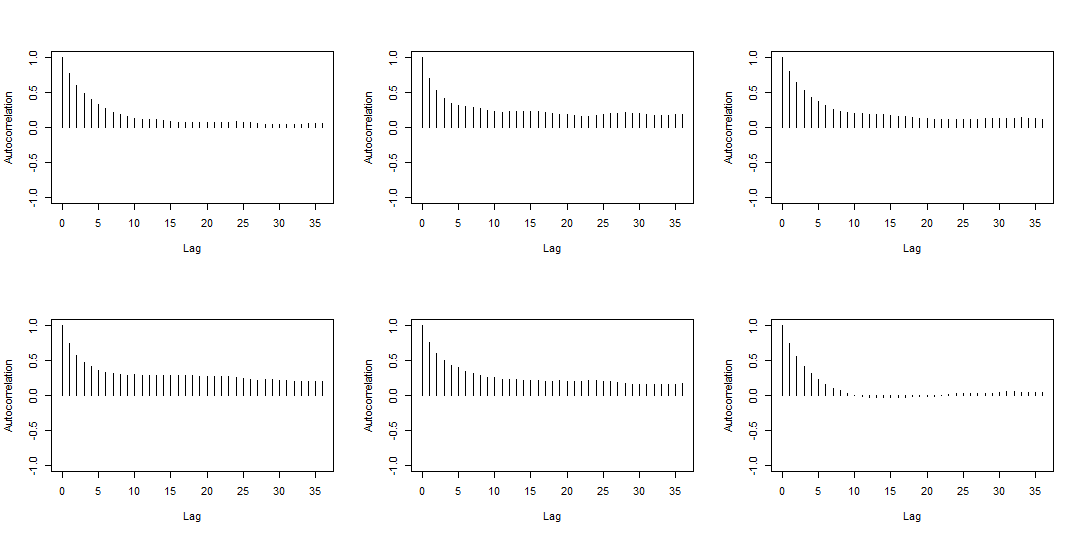
*
