# Supplementary material for: The relationship between biodiversity and wetland cover varies across regions of the conterminous United States
Source: PLoS One. 2020 May 1;15(5):e0232052. doi: 10.1371/journal.pone.0232052 (PMC7194442; doi:10.1371/journal.pone.0232052)
Supplement: Attachment — Submitted filename: Response to Reviewers.docx [file pone.0232052.s002.docx]

Thank you for submitting your work to PLOS ONE. I hereby submit my decision as Academic Editor on PONE-D-19-34337 "The relationship between biodiversity and wetland density varies across regions of the conterminous United States." The two reviewers I invited were generally positive about this submission and I concur with them. Especially, Reviewer 1 was very much satisfied with the work and recommended minor revisions. The second reviewer (a spatial statistician) was a bit concerned with the methodological section, but I believe that you will be able to address the issues raised.

**Response:** *Thank you for your helpful comments and reviews, we believe it substantially helped in the communication of our study especially the methods and results. We have addressed all the comments below and have included supplementary information on our models and model results.*

*In addition to these reviews, we received comments from an internal review from the U.S. Geological Survey (USGS). A notable change that was made from these comments is that we changed “wetland density” to “proportional wetland cover” which indicates the areal proportion of a pixel (100 km^2^) that is covered by wetlands. Wetland density to the USGS reviewer meant the number of individual wetlands per unit area rather than our usage of wetland area per pixel, this led to confusion about the results and inference. Proportional land cover is used by other landscape ecologists (i.e., Gardner et al. 1987, Theobald 2010, 2014) and we believe better explains this variable. This should help with confusion about this variable and hopefully makes the results more accessible to natural resource managers.*

Additionally, I would like you to address the following concerns of mine in the revision:

(1) In the last paragraph of the Intro section, there are a set of predictions (or hypotheses) presented. Please provide some background information as to how you came up with these expectations.

**Response**: *We* i*ncluded an additional background and citations to explain these expectations.*

(2) Fig. 3 -- (a) and (b) are missing. Also, please provide appropriate legends for each panel.

**Response:** *We made these corrections. In addition, we projected the maps in this figure to conform to the coordinate system of the other maps and improved the color palette.*

(3) Fig. 4 -- The legend looks upside down. Please change it so that "1" goes to the bottom and "83" comes to the top.
(4) Figs. 3 and 5 -- The 48 state boundaries are useless in these maps, aren't they? Why don't you present the ecoregion boundaries instead?

**Response**: *We made these changes in figures 3, 4, and 5.*

**Comments to the Author**

**Reviewer 1**

Reviewer comments

I was excited to read this manuscript. The manuscript addresses an interesting topic, the relationship between biodiversity and wetland density across regions of the conterminous United States.
Previous work, such as ‘Status and Trends of Wetlands in the Conterminous United States 2004 to 2009’ (Dahl, 2011) provided baseline information regarding trends in wetland extent and type to facilitate collaborative efforts to assess wetland condition. The geospatial analyses provided insights to regional patterns of wetland loss and indicated that particular regions of the conterminous United States experienced different rates of wetland loss depending on many factors (Dahl, 2011;69). This study digs a bit deeper to address the spatially explicit relationship between wetland density and biodiversity in terms of species distribution of amphibians, birds, mammals, reptiles, and terrestrial endemic species. The authors use National Wetland Inventory to model wetland density for the conterminous United States and the National Land Cover Database to estimate wetland change between 2001 and 2011, and investigate the potential relationship in terms of Bayesian spatial Poisson model. This study builds nicely on past contributions in the field and is a much-anticipated advancement that extends our understanding of the connections between biodiversity and wetland density. In that regard, the manuscript makes a useful contribution to the literature.

**Response:** *Thank you very much, we appreciate the feedback.*

In the introduction (line 65) it might be useful to give more explanation in relation to the purpose of study.

**Response:** *We added a paragraph giving more purpose and context for why it is important to study these relationships and how our study is an advancement in the use of spatially varying data analysis.*

Line (80) Is it due to?

**Response:** *Yes, we made this edit.*

Line (112) Is it hydrologic unit codes (HUC) or hydrologic units (HUC)? How many HUC-2 regions in the conterminous US? What about the next level unit, HUC-4 to analyze the data?

**Response:** *We changed it to hydrologic unit codes and added the number of HUC2 regions in the United States. Model diagnostics of fit for models with HUC2 random effects were better than model diagnostics for models that contained HUC 4 random effects, therefore we continued with the use of HUC 2.*

Line (114) needs a citation or rationale regarding cutoff of 0.7 as an indicator of correlation.

**Response:** *Added two citations regarding the rationale of using 0.7*

Line (296) Consider replacing National Land Cover Database with NLCD.

**Response:** *As this is for a figure caption, we think it should be able to stand alone, therefore we should likely spell out any definition before using an acronym.*

Line (301) Consider replacing Discussion with Discussion and Conclusion.

**Response:** *We made this change.*

In general, the work of Dahl may be relevant:
Dahl, T.E. 2011. Status and trends of wetlands in the conterminous United States 2004 to 2009.
U.S. Department of the Interior; Fish and Wildlife Service, Washington, D.C. 108 pp.
Dahl, T.E. 2006. Status and trends of wetlands in the conterminous United States 1998 to 2004.
U.S. Department of the Interior; Fish and Wildlife Service, Washington, D.C. 112 pp.

**Response:** *Thank you, Dahl, T.E. (2011) is cited in the introduction.*

**Reviewer 2**

I read their manuscript very interestingly. This paper was pretty well written and easy to understand.
However, to publish this manuscript in Plos One, two points should be addressed at the revision.

**Response:** *Thank you for your kind words. You make several excellent points, which we attempt to address below.*

1. In their statistical analysis section, they state that they use Bayesian spatial Poisson model for describing the effect of wetland density on the species of wildlife. However, I think the detailed explanation of the models are missing and it should be required the justification of Bayesian spatially varying Poisson model, especially, the use of the piecewise constant B-spline basis functions. Why we cannot use simpler model? This explanation should be easy in order to make ecologists who have a limited knowledge in statistics understand.

**Response:** *We agree that a simple explanation for the model would be useful for many readers unfamiliar with spatial statistics. Towards this end, we have added a brief explanation of the need for a spatially varying coefficient model to the statistical analysis section of the manuscript, along with a brief explanation of the utility of penalized splines. We also elaborated further on the need for the spatial random effects.*

2. In order to estimate parameter, they use the MCMC sampler. My questions are given followings: (1) What MCMC sampler they use? Generally, implementing the MCMC sampler to the spatial model is not straightforward. Did you really use the simple MH sampler? (2) How many iterations you ran and burned-in? (3) Give some examples of trace plots and autocorrelation plots in your response letter. (4) Provide your computation time. And (5) To show the convergence, try to run the chain multiple times and calculate Gelman-Rubin statistics.

**Response:** *We are happy to provide additional details regarding the MCMC sampling procedure.*

*Regarding (1): Our MCMC sampler consisted mainly of Metropolis steps. The fixed effects, spline basis coefficients, and random effects were sampled in Metropolis steps using adaptive proposal distributions. The prior variance parameters for the spline basis coefficients and random effects were sampled in Gibbs steps. We have created an appendix for our paper that contains the full conditional distributions of the model parameters and a step-by-step implementation of the MCMC sampling algorithm.*

*Regarding (2): We ran our MCMC sampler for 15,000 iterations, with the first 10,000 discarded as burn in, and have added this information to Statistical Analysis section.*

*Regarding (3): We have included trace plots and autocorrelation plots at the end of this document. As there are hundreds of parameters, we have included plots for 6 arbitrarily chosen spline basis coefficients from each of the 5 model fits.*

*Regarding (4): Our computation time was 19.7hrs for the amphibian model, 23.5hrs for the birds model, 23.6hrs for the endemics model, 23.5hrs for the mammals model, and 23.5hrs for the reptiles model. All fits were performed on a Dell Precision 3630 Tower with an Intel® Xeon® E-2186G CPU @3.80 Ghz, 3792 Mhz, with 6 cores and 64 GB of RAM. The reptiles model was fit with no other models running simultaneously, while the other 4 had at least one other model running at the same time, hence the difference in computation times. We have added a sentence regarding the computation time to Statistical Analysis section.*

*Regarding (5): Unfortunately, due to the time and computational intensity of the model fits, internal review requirements, and the fact that we have to fit five separate models, we were unable to perform multiple additional model fits for each model to assess convergence via Gelman-Rubin statistics. However, we are confident in the convergence of our models based on the trace plots that we supplied below. The red line on the trace plots shows the divide before and after the burn-in phase.*

*Plots from the amphibians model (red line indicates the end of the burn-in phase):*

*Plots from the birds model:*

*Plots from the endemics model:*

*Plots from the mammals model:*

*Plots from the reptiles model:*
